# Levodopa treatment and dendritic spine pathology

**DOI:** 10.1002/mds.27172

**Published:** 2017-09-07

**Authors:** Haruo Nishijima, Tatsuya Ueno, Yukihisa Funamizu, Shinya Ueno, Masahiko Tomiyama

**Affiliations:** ^1^ Department of Neurology Aomori Prefectural Central Hospital Aomori Japan; ^2^ Department of Neurophysiology, Institute of Brain Science Hirosaki University Graduate School of Medicine Hirosaki Aomori Japan

**Keywords:** Dendritic spine, dyskinesia, levodopa, Parkinson's disease, synaptic plasticity

## Abstract

Parkinson's disease (PD) is a neurodegenerative disorder associated with the progressive loss of nigrostriatal dopaminergic neurons. Levodopa is the most effective treatment for the motor symptoms of PD. However, chronic oral levodopa treatment can lead to various motor and nonmotor complications because of nonphysiological pulsatile dopaminergic stimulation in the brain. Examinations of autopsy cases with PD have revealed a decreased number of dendritic spines of striatal neurons. Animal models of PD have revealed altered density and morphology of dendritic spines of neurons in various brain regions after dopaminergic denervation or dopaminergic denervation plus levodopa treatment, indicating altered synaptic transmission. Recent studies using rodent models have reported dendritic spine head enlargement in the caudate‐putamen, nucleus accumbens, primary motor cortex, and prefrontal cortex in cases where chronic levodopa treatment following dopaminergic denervation induced dyskinesia‐like abnormal involuntary movement. Hypertrophy of spines results from insertion of alpha‐amino‐2,3‐dihydro‐5‐methyl‐3‐oxo‐4‐isoxazolepropanoic acid receptors into the postsynaptic membrane. Such spine enlargement indicates hypersensitivity of the synapse to excitatory inputs and is compatible with a lack of depotentiation, which is an electrophysiological hallmark of levodopa‐induced dyskinesia found in the corticostriatal synapses of dyskinetic animals and the motor cortex of dyskinetic PD patients. This synaptic plasticity may be one of the mechanisms underlying the priming of levodopa‐induced complications such as levodopa‐induced dyskinesia and dopamine dysregulation syndrome. Drugs that could potentially prevent spine enlargement, such as calcium channel blockers, N‐methyl‐D‐aspartate receptor antagonists, alpha‐amino‐2,3‐dihydro‐5‐methyl‐3‐oxo‐4‐isoxazolepropanoic acid receptor antagonists, and metabotropic glutamate receptor antagonists, are candidates for treatment of levodopa‐induced complications in PD. © 2017 The Authors. *Movement Disorders* published by Wiley Periodicals, Inc. on behalf of International Parkinson and Movement Disorder Society.

L‐3,4‐dihydroxyphenylalanine (levodopa) is the most effective treatment for alleviating motor symptoms of PD.^1^, ^2^ However, chronic oral administration of levodopa induces motor complications such as levodopa‐induced dyskinesia (LID) and neuropsychiatric complications such as dopamine dysregulation syndrome (DDS).^3^, ^4^ The pathophysiology of motor complications associated with PD, particularly LID, has been extensively investigated.^5^ Importantly, it has been shown that LID is associated with abnormal plasticity of corticostriatal synapses.^6^, ^7^ There is also increasing evidence that direct pathway neurons and D1 dopamine receptors play a crucial role in LID.^8^, ^9^, ^10^, ^11^, ^12^, ^13^

Dendritic spines form the postsynaptic part of excitatory synapses. Increasing evidence suggests that spines dynamically change their density and morphology, and that these changes are deeply involved in synaptic plasticity.^14^ Spine head size is reported to increase following the induction of long‐term potentiation (LTP), and to decrease with long‐term depression.^14^, ^15^, ^16^, ^17^ An electrophysiological study revealed that miniature excitatory postsynaptic current (mEPSC) amplitude is correlated with dendritic spine size, whereas mEPSC frequency is correlated with spine density.^18^ Thus, the density and morphology of dendritic spines determine synaptic strength.^19^, ^20^ It has been demonstrated that the dopamine‐dependent mechanism is a key step in learning, while dopaminergic modification is deeply involved with synaptic plasticity at axospinous synapses in the striatum and cortex.^21^, ^22^, ^23^, ^24^, ^25^ A recent study demonstrated that dopamine promotes spine enlargement only during a narrow time window after the glutamatergic inputs at the level of single dendritic spines.^26^ The timing and duration of dopamine signals are important in controlling synaptic plasticity,^24^ and they are dramatically distorted with levodopa treatment in PD patients.^24^ This distortion is likely to underlie LID. In addition to the above‐mentioned synaptic plasticity at the level of single dendritic spines, studies by Surmeier's research group^22^, ^23^ suggest that homeostatic plasticity is important for changes in dendritic spine density and morphology. Normal neurons are homeostatic, and homeostatic mechanisms attempt to bring neuronal activity back to the normal level when there are perturbations in synaptic or intrinsic properties that make neurons spike more or less than their set point.^23^ In this type of plasticity, dopamine receptors control intrinsic excitability^27^, ^28^ through the modulation of ion channels,^22^, ^23^, ^24^ which can be therapeutic targets for PD and LID.

Autopsy examinations using Golgi impregnation have revealed decrements of dendritic spine density of striatal spiny projection neurons (SPNs) of PD patients receiving levodopa,^29^, ^30^, ^31^ indicating that either the parkinsonian state itself and/or antiparkinsonian drug treatment affect dendritic spine pathology. In the current review, we summarize recent advances in research into the changes of spine morphology associated with levodopa treatment in animal models of PD.

## Spiny Projection Neurons in the Caudate‐Putamen

Projection neurons in the caudate‐putamen (CPu) are SPNs that form the first segment of the direct and indirect pathways of basal ganglia circuits. SPNs of the direct pathway (dSPN), expressing D1 dopamine receptors, project to the internal segment of the globus pallidus and the substantia nigra pars reticulata. SPNs of the indirect pathway (iSPN), expressing the D2 dopamine receptors, project to the external segment of the globus pallidus (GPe).^32^ However, recent studies revealed coexpression of D1 and D2 receptors and collaterals, which bridge the direct and indirect pathways.^33^, ^34^ The distinction between the 2 types of SPNs is less clear than traditionally thought.

SPNs in the CPu receive glutamatergic inputs from the cortex and dopaminergic inputs from the substantia nigra pars compacta (SNpc). Glutamatergic terminals from the cortex synapse onto the heads of dendritic spines of SPNs. Dopaminergic terminals synapse onto the dendrites broadly, including the necks of spines or dendritic shafts.^35^, ^36^ Because glutamatergic and dopaminergic inputs are integrated in the spines, frequent interactions are likely to occur between inputs in spines. This leads to synaptic plasticity and spine pathological changes, resulting from dopaminergic denervation and further levodopa treatment.

### Dendritic Spine Loss by Dopaminergic Denervation

In a rat model of PD created by 6‐hydroxydopamine (6‐OHDA) injection to the medial forebrain bundle (MFB), dendritic spines of SPNs in the dopamine‐denervated CPu are reduced in density.^37^ In addition to spine density, the number of asymmetric synapses was also found to be reduced in the lesioned CPu, suggesting that spine decrement reflects a decrement of excitatory corticostriatal synapses.^38^ Other studies also demonstrated the loss of dendritic spines of SPNs in patients with PD,^30^, ^31^ 1‐methyl‐4‐phenyl‐1,2,3,6‐tetrahydropyridine (MPTP)‐treated parkinsonian monkeys,^39^ and mouse models of PD.^27^, ^28^, ^40^, ^41^, ^42^

Previous studies have produced conflicting results regarding which types of SPN (ie, dSPNs or iSPNs) undergo alteration of spine density after dopaminergic denervation. In bacterial artificial chromosomes (BACs), transgenic mice expressing enhanced green fluorescent protein under the control of cell type–specific promoters (D1 receptor expressing dSPNs or D2 receptor expressing iSPNs), dopamine depletion by 6‐OHDA injection to the MFB was found to induce a spine decrement in iSPNs, but not dSPNs.^43^ In reserpine‐treated PD model mice, it was found that iSPNs lost dendritic spines, whereas dSPNs did not.^43^ Shen and colleagues^44^ reported substantial pruning of spines and glutamatergic synapses in iSPNs, leaving dSPNs intact in PD model mice. A study of MPTP‐treated PD model monkeys reported the loss of D2 receptor positive asymmetric synapses in the CPu, but not D1 receptor positive synapses.^45^ In addition, a study of 6‐OHDA‐lesioned hemiparkinsonian rats in our lab revealed that iSPNs, but not dSPNs, lost dendritic spines.^46^

Some studies have demonstrated dendritic spine pruning in both types of SPNs in the CPu. In MPTP‐treated PD model monkeys, spine analysis using immunocytochemistry and electron microscopy revealed a significant spine density reduction in both D1 immunoreactive and D1 immunonegative spines.^39^ Toy and colleagues^40^ reported that both dSPNs and iSPNs lost their dendritic spines, and treadmill exercise restored the reduction in a MPTP‐treated mouse model of PD. Suarez and colleagues^27^, ^28^ demonstrated that dopamine depletion by 6‐OHDA injection to the striatum reduces spine density in both dSPNs and iSPNs using both D1‐tomato and D2‐enhanced green fluorescent protein BAC transgenic mice. Their results were reproduced in a study using BAC transgenic mice, revealing that striatal dopamine depletion induced by 6‐OHDA injection to the MFB led to dendritic spine loss in D1 receptor positive, D2 receptor positive, and D1 and D2 receptor positive SPNs.^42^

Thus, it remains unclear which type of SPN in the CPu lose dendritic spines after dopamine depletion. Discrepancies between studies may be related to differences between species (rodents or primates), dopamine‐denervation methods (6‐OHDA injection, acute or chronic MPTP treatment, or reserpine treatment), or image analysis (light microscopy, confocal laser microscopy, 2‐photon excitation fluorescence microscopy, or electron microscopy). These methodological differences are precisely discussed by Moratalla and colleagues.^47^ Among these factors, dopamine‐denervation methods may have the strongest impact. In the acute phase of dopaminergic denervation induced by 6‐OHDA in rodents, some authors find that iSPNs are more sensitive than dSPNs,^41^, ^43^ whereas others find that both i‐ and dSPN lose spines.^27^, ^28^, ^42^ On the other hand, in a chronic phase of repetitive MPTP treatment for animals, both types of neurons lose dendritic spines.^39^, ^40^

### Effects of Dopaminergic Denervation on Dendritic Spine Size

A small number of studies have examined dendritic spine size in animal models of PD and LID. One study using 3‐dimensional electron microscopy demonstrated enlargement of dendritic spines in the CPu of MPTP‐treated PD model monkeys.^48^ Naskar and colleagues^49^ also showed spine head enlargement after dopaminergic denervation in the CPu of PD model mice created by acute MPTP administration. In contrast, a study in our lab revealed no significant changes in spine head size in the CPu of 6‐OHDA‐lesioned PD model rats.^46^, ^50^ Suarez and colleagues^28^ reported similar findings; no change in the head diameter of mushroom and thin spines in both d‐ and iSPNs in parkinsonian mice. To date, few studies have addressed this issue. It remains unclear whether dopaminergic denervation alone induces hypertrophy of dendritic spines of SPNs.

Fieblinger and colleagues^41^ did not report changes in spine size, but have reported cell‐type specific alterations in mEPSC amplitude following 6‐OHDA lesions in mice. mEPSC amplitude increased in iSPNs and decreased in dSPNs. It was hypothesized that the loss of D2 receptor signaling drives LTP in iSPNs and long‐term depression in dSPNs, which can be associated with potential alterations in dendritic spine size with dopaminergic denervation, whereas other authors reported a loss of long‐term depression in both types of SPN.^51^

### Effects of Levodopa Treatment on Dendritic Spine Pathology in Dopamine‐Denervated Caudate‐Putamen

A previous study reported that synaptic dopamine concentration was markedly reduced in the dopamine‐denervated CPu of 6‐OHDA‐lesioned PD model rats.^52^, ^53^, ^54^ When levodopa was systemically given, dopamine concentration dramatically fluctuated.^55^ In addition, this fluctuation of dopamine is reported to be more prominent in rats expressing LID‐like abnormal behavior.^56^ This abnormal dopamine metabolism results in electrophysiological abnormalities in corticostriatal synapses on dSPNs in the rat model of LID.^6^ In the normal CPu, high‐frequency stimulation of the corticostriatal pathway induces LTP in SPNs that can be reversed with low‐frequency stimulation (depotentiation). In the denervated CPu of 6‐OHDA‐lesioned rats, high‐frequency stimulation cannot induce LTP in corticostriatal synapses. Modest levodopa treatment can restore LTP in the dopamine‐denervated CPu, and depotentiation can revive.^6^ LTP is observed in rats with abnormal involuntary movements induced by repetitive levodopa treatment (ie, in the rat model of LID), but low‐frequency stimulation fails to induce depotentiation. This lack of depotentiation has been observed in dSPNs, but not iSPNs. Thus, the lack of depotentiation in the corticostriatal synapses of dSPNs may be the electrophysiological characteristics of abnormal synaptic plasticity in the model of LID.^6^ Spine function changes in parallel with spine morphology,^15^ suggesting that the lack of depotentiation may be linked with morphological changes of dendritic spines in SPNs, particularly dSPNs. Recent studies of striatal synaptic plasticity have shown that LTP can be induced not just by abnormal high‐frequency stimulation in the absence of extracellular glutamate, but by spike‐timing dependent plasticity protocols in physiological artificial cerebrospinal fluid.^7^, ^57^ Shen and colleagues^7^ reported that application of a D1 receptor antagonist or stimulation of cholinergic interneurons and M4 muscarinic receptors completely restored depotentiation in the mouse PD model. In levodopa‐treated mice, striatal dopamine levels were elevated, leading to sustained D1 receptor stimulation, and depotentiation in dSPNs only occurred when the D1 receptor signaling stopped, indicating that the intracellular machinery underlying depotentiation may have remained intact.^7^ Thus, the lack of depotentiation can be associated with LID expression. However, it remains unclear whether the lack of depotentiation is also associated with priming of LID.

Scholz and colleagues^45^ reported that dopaminergic denervation reduced D2 receptor positive asymmetrical synapses and increased D1 receptor positive synapses using electromicroscopic examination in the CPu of MPTP‐treated monkeys. Levodopa treatment restored the density of both synapse types to control levels with LID expression.^45^ Recently (from 2013 to 2016), 4 research groups, including our own, published studies on dendritic spine pathology in the CPu of animal models of LID.^27^, ^28^, ^41^, ^46^, ^50^, ^58^ There are some parallels and some inconsistencies between the findings of these studies, as described next.

#### Levodopa‐Induced Dyskinesia and Dendritic Spine Density

We examined drebrin immunoreactivity in the CPu of a rat model of PD created by 6‐OHDA injection to the MFB.^50^ Drebrin is a neuron‐specific F‐actin binding protein exclusively localized at the excitatory synapse. Thus, drebrin immunoreactivity can be considered to represent dendritic spines. The number of drebrin immunoreactive dots in the CPu decreased after dopaminergic denervation, and the decrement was not restored with levodopa treatment.^50^ It should be noted that drebrin immunoreactivity may fail to detect thin spines. For more detailed analyses of dendritic spine pathology, we also examined spine morphology of dSPNs and iSPNs separately using a retrograde labeling method, microinjection of fluorescent dye, and confocal microscopy.^46^ Dopaminergic denervation alone did not cause any significant changes in the spine density of dSPNs, but reduced the density of iSPNs. Levodopa treatment with induction of LID decreased the spine density of dSPNs.

Zhang and colleagues^58^ demonstrated, using immunohistochemical and electron microscopic examination, that vesicular glutamate transporter 1 (VGluT1)‐positive synapses (i.e., corticostriatal synapses) were reduced in PD model rats and restored in levodopa‐treated LID model rats. The researchers also demonstrated, with Golgi impregnation and light microscopy, that striatal dendritic spine density was decreased in PD model rats and restored in LID model rats.^58^ They did not differentiate dSPNs from iSPNs.

Suarez and colleagues^27^ used BAC transgenic mice lesioned with 6‐OHDA injection to the striatum. Dendritic spines of D1 receptor positive or D2 receptor negative neurons (putative dSPNs) and D1 receptor negative or D2 receptor positive neurons (putative iSPNs) were examined using confocal microscopy with single‐cell microinjections of fluorescent dye. They revealed that spine density was reduced in both dSPNs and iSPNs in the PD model mouse. Levodopa treatment induced LID and restored the spine density of iSPNs.^27^, ^28^

Fieblinger and colleagues^41^ also examined BAC transgenic mice lesioned with 6‐OHDA injection to the MFB using single‐cell microinjection and 2‐photon microscopy. The results demonstrated that the dendritic spine density of iSPNs was reduced by dopaminergic denervation and restored by high‐dose levodopa treatment with the development of LID. In addition, high‐dose levodopa treatment reduced dendritic spine density in dSPNs.^41^

In addition to the previously mentioned studies examining LID model rodents, Naskar and colleagues^49^ reported the effects of levodopa treatment at low dose without induction of LID in PD model mice created by acute MPTP administration. The results revealed that dopamine denervation after MPTP treatment induced a reduction of spine density and parkinsonian motor symptoms. Low‐dose levodopa treatment did not restore reduced spine density or improve the motor symptoms. Coadministration of melatonin with low‐dose levodopa improved dendritic spine density and motor performance. Although the study did not differentiate dSPNs and iSPNs,^49^ the results indicate that spine restoration in iSPNs might be associated with the improvement of motor performance, but not LID.

Taken together, these studies suggest that dopamine depletion in hemiparkinisonian rodent models induces spine loss of iSPNs, which can be restored with levodopa treatment. Furthermore, levodopa treatment with the development of LID may reduce spine density in dSPNs. These changes of dendritic spine density are summarized in Table 1.

**Table 1:** Changes of dendritic spine density and spine head size in spiny projection neurons in the caudate‐putamen after dopaminergic denervation and levodopa treatment

Animal model	Dopaminergic denervation alone		Dopaminergic denervation plus levodopa treatment (levodopa‐induced dyskinesia model)		Levodopa treatment in normal caudate‐putamen	
Neuron type	dSPN	iSPN	dSPN	iSPN	dSPN	iSPN
Spine density	→^41^, ^43^, ^44^, ^46^ or ↓^27^, ^28^, ^39^, ^40^, ^42^	↓^27^, ^28^, ^39^, ^40^, ^41^, ^42^, ^43^, ^44^, ^46^	↓^41^, ^46^	↑(recovery to normal level) 27, 28, 41	→^28^, ^46^	→^28^, ^46^
Spine head size	→^28^, ^46^, ^50^ or ↑^48^, ^49^, ^a^	→^28^, ^46^, ^50^ or ↑^48^, ^49^, ^a^	↑^28^, ^46^, ^50^	→^28^	↑^46^	?

dSPN, spiny projection neurons forming the direct pathway; iSPN, spiny projection neurons forming the indirect pathway; ↑, increment of density or size; →, no change; ↓, decrement of density or size; ?, data not available.

a Two studies showing an increment of size, which did not differentiate dSPNs and iSPNs.

#### Levodopa‐Induced Dyskinesia and Dendritic Spine Size

**Figure 1:**
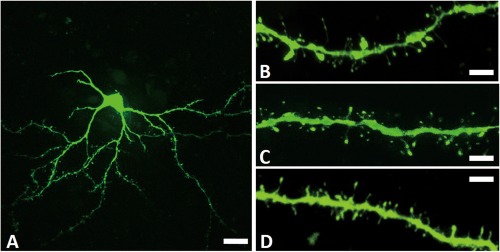
A soma, dendrites, and dendritic spines of striatal spiny projection neurons forming a direct pathway in rats. These are maximum intensity projection images constructed from stacked data acquired using confocal microscopy. The neurons and dendrites are visualized by fluorescent dye, Lucifer yellow. (**A**) A soma and dendrites in a rat model of levodopa‐induced dyskinesia. Scale bar = 20 μm. (**B**) Dendritic spines in a rat model of levodopa‐induced dyskinesia. Some of the dendritic spines exhibit markedly enlarged heads. Scale bar = 5 μm. (**C**) Dendritic spines in a normal rat. Scale bar = 5 μm. (**D**) Dendritic spines in a Parkinson's disease model rat without levodopa treatment. There were no significant changes in spine size when compared with that of a normal rat. Scale bar = 5 μm.

Few reports have examined the relationship between dendritic spine size and LID. In 6‐OHDA‐lesioned PD model rats, levodopa treatment with the development of LID increased mushroom‐type spines in striatal SPNs.^58^ We previously demonstrated that levodopa treatment enlarged striatal drebrin immunoreactive dots (putative mushroom‐type dendritic spines) in LID model rats.^50^ Moreover, in another study, we found spine head enlargement in dSPNs of the CPu in the same LID model rats using fluorescent dye microinjection and confocal microscopy^46^ (Fig. 1). However, some SPNs projecting to the GPe also exhibited enlarged dendritic spines. We used normal nontransgenic Wistar rats, differentiating between dSPNs and iSPNs using retrograde labeling. Using this labeling method, SPNs projecting to the GPe might include dSPNs labeled via collaterals to the GPe. Thus, SPNs with enlarged spines projecting to the GPe might be dSPNs. Based on these findings, we concluded that levodopa treatment with LID expression in PD model rats appears to enlarge dendritic spines of dSPNs.

A recent study by Suarez and colleagues^28^ revealed detailed striatal dendritic spine morphology associated with LID using BAC transgenic mice with 6‐OHDA‐lesioning in the striatum, fluorescent dye microinjection and confocal microscopy, and electron microscopy. The electron microscopy revealed that dSPNs exhibited prolonged spine length and postsynaptic density length after levodopa treatment with expression of LID. Dopaminergic denervation alone did not change spine length or postsynaptic density length in dSPNs. In iSPNs, postsynaptic density length decreased in LID model mice. Fluorescent dye microinjection and confocal microscopy revealed that the density of mushroom‐type and thin spines does not change in dSPNs in the LID model, but is restored in iSPNs. This finding conflicts with Zhang and colleagues'^58^ report of increased mushroom‐type spines with LID. Regarding the size of each spine, they showed no significant changes in spine volume or length in dSPNs in PD model rats and a significant increment of volume and length of mushroom‐type spines in dSPNs in LID model rats. Spine head volume in the iSPN of the LID model did not show significant changes.^28^ The study also demonstrated that the firing rate increased in both dSPNs and iSPNs with dopaminergic denervation. The rate was decreased to control levels in iSPNs with further levodopa treatment, but remained high in dSPNs even after levodopa treatment. These results also provide evidence for an association between LID expression and spine head enlargement. The changes of dendritic spine head size in CPu are summarized in Table 1.

These findings suggest that spine loss and spine head enlargement in dSPN may be essential structural characteristics in LID models. The animals in studies examining dendritic spine pathology with LID, including ours,^41^, ^46^, ^58^ were euthanized more than 12 hours after levodopa treatment when they did not display any dyskinesias, whereas the animals in the other studies^27^, ^28^, ^45^ were euthanized 1 hour after levodopa administration. The structural characteristics in the former studies are likely to have represented the priming of LID and in the latter studies the expression of LID. The differences in the timing of euthanasia may have contributed to the discrepancy in the findings of previous studies.

Spine head enlargement results from insertion of alpha‐amino‐3‐hydroxy‐5‐methyl‐4‐isoxazolepropionic acid (AMPA) receptors in the postsynaptic membrane.^14^, ^17^ Hypertrophic spines are supersensitive for glutamatergic inputs.^14^, ^17^ This structural change is consistent with the characteristic electrophysiological synaptic dysfunction (ie, the lack of depotentiation) in dSPNs in the LID model.^6^

### Effects of Levodopa Treatment on Dendritic Spine Pathology in the Normal Caudate‐Putamen

In the study by Suarez and colleagues,^28^ levodopa treatment for sham‐lesioned mice did not affect spine density in either dSPNs or iSPNs. However, the researchers did not examine spine volume. Similarly, our study showed no changes in spine density in either SPN type after levodopa treatment in normal rats.^46^ However, spine head enlargement has been found with levodopa treatment in normal rats without induction of LID.^46^ Striatal dopamine concentration has been found to fluctuate after levodopa administration, even in normal rat CPu.^59^ Repetitive high doses of levodopa may induce dyskinesia, even in normal monkeys.^60^ Thus, levodopa potentially changes synaptic plasticity even in the normal CPu. However, the enlargement of spines in the normal CPu was not accompanied by LID in our experiments.^46^, ^50^ Accordingly, the enlargement of dendritic spines may be necessary but not sufficient to cause LID. These observations suggest that dendritic spine enlargement, concomitant with spine pruning in dSPNs, may be essential for the priming of LID.^46^

## Spiny Projection Neurons in the Nucleus Accumbens

The nucleus accumbens (NAc), located in the ventral striatum, plays an essential role in the reward system, being associated with various neuropsychiatric disorders including drug addiction and psychological symptoms in PD.^61^ Most neurons in the NAc are SPNs. DDS is also induced by levodopa treatment for PD patients. DDS appears to result from marked fluctuation of synaptic dopamine levels in the NAc after levodopa administration^62^ as in the CPu of LID patients.^63^ Almost all DDS patients display LID, suggesting that DDS and LID are likely to share pathogenic mechanisms.^64^

In a study of PD model rats created by 6‐OHDA injection to the SNpc, modified Golgi‐Cox staining revealed a decrease in spine density in the dopamine‐denervated NAc.^65^ We examined drebrin immunoreactive dots (putative dendritic spines) in the NAc of PD model rats created by 6‐OHDA injection to the MFB. We failed to find changes in the density and size of drebrin immunoreactive dots in the PD model rats.^50^ In an LID model, we found that dot density decreased, and the size of dots increased in the NAc.^50^ It should be noted that drebrin immunoreactive dots may not reflect all dendritic spines. Thus, we conducted a detailed examination using single‐cell microinjection of fluorescent dye in PD and LID model rats.^66^ Dopaminergic denervation alone decreased dendritic spine density both in the core and shell. Levodopa treatment after dopaminergic denervation induced LID, restoring spine density and enlarging spines both in the core and in the shell, indicating hypersensitivity of SPNs in the NAc for excitatory inputs. Levodopa treatment in normal rats was found to enlarge spines of SPNs in the shell but not in the core of the NAc, without changes in spine density.^66^ Previous studies have shown that the shell of the NAc is associated with the development of addiction, and the core is relevant to the long‐term execution of learned addiction‐related behaviors.^67^, ^68^, ^69^ Our results indicate that spine enlargement in the shell of the NAc is linked with the initial onset of levodopa‐induced abnormal behaviors, whereas spine enlargement in the core is associated with persistent behavioral changes induced by levodopa treatment. Thus, LID model rats appear to undergo morphological changes of SPNs in the NAc as well as the CPu, suggesting that common synaptic plasticity mechanisms underlie the LID and DDS. The changes of dendritic spine density and spine volume in the NAc are summarized in Table 2.

**Table 2:** Changes of dendritic spine density and spine head size in spiny projection neurons in the nucleus accumbens after dopaminergic denervation and levodopa treatment

Animal model	Dopaminergic denervation alone		Dopaminergic denervation plus levodopa treatment (levodopa‐induced dyskinesia model)		Levodopa treatment in normal nucleus accumbens	
Location of SPN	Core	Shell	Core	Shell	Core	Shell
Spine density	↓^65^, ^66^	↓^65^, ^66^	↑ (recovery to normal level)^66^	↑ (recovery to normal level)^66^	→^66^	→^66^
Spine head size	→^66^	→^66^	↑^50^, ^66^	↑^50^, ^66^	→^66^	↑^66^

SPN, spiny projection neuron; ↑, increment of density or size; →, no change; ↓, decrement of density or size.

Increasing evidence suggests that morphological changes in SPN spines in the NAc are related to addictive behavior in cocaine use,^70^ which increases synaptic dopamine levels.^71^ SPN spines in the NAc become enlarged in the withdrawal phase of cocaine exposure.^72^ Thus, the enlargement of SPN spines in the NAc may account for the psychological dependence of cocaine addiction.^72^ Taken together, SPN spine enlargement in the NAc of the LID model rat may represent a pathological hallmark associated with behavioral dependence on levodopa in DDS. However, in the previously discussed study, we investigated SPN spines in an LID model, but not a DDS model. It would be valuable for future studies to create a distinct DDS model to address this limitation.

## Pyramidal Neurons in the Primary Motor Cortex

The primary motor cortex (M1) is a motor function center that receives dopaminergic input from the ventral tegmental area.^73^ PD patients are reported to lose dopaminergic neurons in the ventral tegmental area, although the loss is modest when compared with that of the SNpc.^74^ Rodent studies have demonstrated the following 2 types of pyramidal neurons in the cortex: intratelencephalic (IT)‐type neurons that preferentially innervate to dSPNs and express D1 dopamine receptors, and pyramidal tract (PT)‐type neurons that preferentially innervate to iSPNs and express D2 dopamine receptors.^75^ However, a recent study reported the controversial finding that both dSPNs and iSPNs responded to both IT‐ and PT‐type neuron activation, arguing that these cortical networks similarly innervate both striatal projection neuron types.^76^

Abnormal synaptic plasticity in the M1 cortex has been demonstrated in a clinical study using transcranial magnetic stimulation in PD patients.^77^ Transcranial magnetic stimulation has been used to test the integrity of the corticospinal tract and has now been applied in various examination and therapeutic settings based on spike‐timing plasticity mechanisms.^78^, ^79^ Paired associative stimulation was found to increase the size of motor‐evoked potentials in controls, but did not affect PD patients without levodopa treatment. Levodopa treatment without the induction of dyskinesia revived the response.^77^ Another study applied transcranial magnetic stimulation in PD patients with LID.^80^ In controls, high‐frequency stimulation induced LTP‐like elevated motor‐evoked potentials, which were reversed by low‐frequency stimulation (ie, “depotentiation‐like” effects). In PD patients with LID, the depotentiation‐like effect was lost.^80^ This lack of synaptic depotentiation‐like effects in the brain is similar to the lack of depotentiation demonstrated in dSPNs in the rat model of LID.^6^ To date, few studies have examined morphological changes of dendritic spines of pyramidal neurons in the M1 cortex in PD and LID models.

We first examined dendritic spines of IT‐type pyramidal neurons in the M1 cortex in PD and LID model rats created by 6‐OHDA injection to the MFB.^81^ 6‐OHDA injection to the MFB induces dopamine denervation not only in the striatum but also in the M1 cortex^81^ and prefrontal cortex (PFC).^82^ Single‐cell microinjection of fluorescent dye and confocal microscopy examination of IT‐type neurons in the M1 revealed no changes of dendritic spine density, but slight increases in spine head size in a PD model. Further spine head enlargement was observed in an LID model. Levodopa treatment in normal rats has no effect on spine head size.^81^ In addition, we monitored mEPSCs in IT‐type pyramidal neurons in M1 cortex using a whole‐cell patch clamp method. The amplitude of mEPSCs was unchanged in the PD model, but increased in the LID model, whereas the firing rate was unchanged in the both models.^81^ These results suggest that IT‐type pyramidal neurons in the M1 cortex became hypersensitive in the LID model. We then examined PT‐type pyramidal neurons in the same PD and LID model rats.^83^ Dopaminergic denervation alone did not change spine density or spine head size. Spine density mildly increased and spine heads became enlarged in the LID model. Levodopa treatment for normal rats induced no LID‐like behavior or spine head enlargement, but induced an increase in spine density. PT‐type neurons also exhibited structural alterations with LID in the rodent PD model. Thus, both IT‐type and PT‐type neurons in the M1 cortex displayed enlargement of the dendritic spines, which may be one of the pathophysiological mechanisms underlying LID. Changes of dendritic spine density and spine head size in M1 cortex are summarized in Table 3.

**Table 3:** Changes of dendritic spine density and spine head size in pyramidal neurons in the primary motor cortex after dopaminergic denervation and levodopa treatment

Animal model	Dopaminergic denervation alone		Dopaminergic denervation plus levodopa treatment (levodopa‐induced dyskinesia model)		Levodopa treatment in normal primary motor cortex	
Type of pyramidal neurons	IT type	PT type	IT type	PT type	IT type	PT type
Spine density	→^81^ or ↓^86^, ^a^	→^83^ or ↓^86^, ^a^	→^81^	↑^83^	→^81^	↑^83^
Spine head size	↑^81^	→^83^	↑^81^	↑^83^	→^81^	→^83^

IT, intratelencephalic; PT, pyramidal tract; ↑, increment of density or size; →, no change; ↓, decrement of density or size.

a Study showing a decrement of density that did not differentiate IT‐ and PT‐type neurons.

In our studies, neither IT‐type nor PT‐type pyramidal neurons in M1 cortex exhibited spine density alterations after dopaminergic denervation alone. This finding is consistent with previous studies.^84^, ^85^ In contrast, spine density was reduced in the M1 cortex in MPTP‐treated PD model mice.^86^ Discrepancies between the studies may be due to differences in dopamine‐denervation methods (local 6‐OHDA injection and systemic MPTP administration). MPTP have widespread effects on neural tissue, affecting not only the dopaminergic system but also other monoamine systems.^87^

LID may be associated with hypersensitivity of neurons not only in the CPu and NAc, but also in the M1 cortex. The enlargement of dendritic spines in the M1 cortex are compatible with abnormal cortical plasticity and a lack of depotentiation‐like effects demonstrated in clinical research in patients with LID.^80^ To our knowledge, our study is the only report of changes in spine density and morphology in the M1 cortex associated with LID. Further research is needed to confirm our findings.

## Pyramidal Neurons in the Prefrontal Cortex

The PFC is associated with attention, impulse control, learning, and memory. PFC dysfunction is related to various neuropsychiatric disorders, including drug addiction and impulse control disorder.^88^, ^89^, ^90^, ^91^ The PFC forms part of the reward system, receiving excitatory inputs from the thalamus, hippocampus, and amygdala and dopaminergic inputs from the ventral tegmental area.^61^ In the PFC, 80% to 90% of neurons are excitatory pyramidal cells.^92^, ^93^ As in the M1 cortex, there are PT‐type and IT‐type neurons in the PFC.^75^, ^92^, ^94^ Levodopa‐related complications in PD patients, such as LID and DDS, are considered to be partially caused by dysfunction of prefrontal neurons.^95^ A clinical study using magnetic resonance imaging reported that the thickness of the PFC was increased in PD patients with LID when compared with those without LID.^96^ In rats with dopaminergic denervation by 6‐OHDA injection to the SNpc, pyramidal cells in the medial PFC have been observed to exhibit an elevated firing rate, suggesting the acquisition of hyperactivity in these cells.^97^, ^98^

A small number of studies have examined microscopic morphological changes of prefrontal neurons associated with the physiological and clinical abnormalities described previously. Solis and colleagues^65^ found reduced spine density of neurons in the medial PFC in PD model rats created by 6‐OHDA injection to the SNpc. Moreover, in a study of MPTP‐treated monkeys, the number of asymmetric excitatory synapses was found to be reduced in dorsolateral PFC.^99^ We recently examined dendritic spine density and spine head volume of PT‐type prefrontal neurons in PD and LID model rats.^82^ Dopaminergic denervation with 6‐OHDA lesioning in the MFB induced no changes in spine density or spine head volume of pyramidal neurons in the PFC. Levodopa treatment in dopamine‐denervated rats decreased spine density and increased spine head volume with LID expression. Levodopa treatment in normal rats had no effect on spine morphology. Our results did not reveal spine loss after dopaminergic denervation alone, inconsistent with previous studies. Because we only examined PT‐type neurons distinguished by retrograde labeling, it is possible that PT‐type neurons do not decrease spine density, whereas IT‐type neurons decrease spine density after dopaminergic denervation. Changes in dendritic spine density and spine head size in the PFC are summarized in Table 4.

**Table 4:** Changes of dendritic spine density and spine head size in pyramidal neurons in the prefrontal cortex after dopaminergic denervation and levodopa treatment

Animal model	Dopaminergic denervation alone		Dopaminergic denervation plus levodopa treatment (levodopa‐induced dyskinesia model)		Levodopa treatment in normal prefrontal cortex	
Type of pyramidal neurons	IT type	PT type	IT type	PT type	IT type	PT type
Spine density	↓^65^, ^99^, ^a^	→^82^	?	↓^82^	?	→^82^
Spine head size	?	→^82^	?	↑^82^	?	→^82^

IT, intratelencephalic; PT, pyramidal tract; ↑, increment of density or size; →, no change; ↓, decrement of density or size; ?, data not available.

a Two studies showing a decrement of density with dopamine denervation alone, which did not differentiate IT‐ and PT‐type neurons.

Our findings, spine density reduction and spine head hypertrophy in PT‐type neurons of the PFC in LID model rats,^82^ may be the structural basis of abnormal synaptic transmission in the PFC with LID. In the PFC of PD model rats, as in the striatum,^55^ systemic levodopa administration induces nonphysiological fluctuations of extracellular dopamine concentration,^100^, ^101^ which may be responsible for dendritic spine loss and hypertrophy. In our study, levodopa treatment in the normal PFC induced no changes in spine density or spine head volume, and the rats expressed no LID‐like involuntary movements. Levodopa, unlike cocaine and morphine, may not induce synaptic abnormalities in the PFC in the normal state where dopaminergic innervation is preserved. To our knowledge, the effects of levodopa treatment on dendritic spine morphology in the PFC have only been investigated by our research group. More studies are warranted to confirm our findings.

## Closing Remarks

Dopaminergic denervation and following levodopa treatment strongly affects dendritic spine density and morphology. Levodopa treatment in the normal brain also exerts several effects. Repetitive levodopa treatment in animal models of PD sufficient to induce LID has been found to increase spine head size in all brain regions investigated to date. This enlargement is consistent with the electrophysiological hallmark of LID, the lack of depotentiation. Spine enlargement may be the structural basis of levodopa‐induced complications in PD patients, such as LID and DDS.

Dopamine‐induced synaptic or homeostatic plasticity is associated with activities of *N*‐methyl‐d‐aspartate and AMPA receptors and modulation of ion channels including L‐type Ca^2+^ channels.^22^, ^24^ Neuronal dendrites are richly endowed with various voltage‐dependent ion channels that shape synaptic responses.^23^ Drugs that block these receptors or ion channels potentially prevent spine loss and spine head enlargement and might be beneficial for the treatment of LID and DDS. A number of studies have examined potential drug treatments,^5^ including Ca^2+^ blockers,^102^, ^103^ *N*‐methyl‐d‐aspartate receptor antagonists,^104^, ^105^, ^106^ AMPA receptor antagonists,^107^, ^108^, ^109^, ^110^, ^111^, ^112^ and metabotropic glutamate receptor antagonists.^113^, ^114^, ^115^, ^116^, ^117^, ^118^, ^119^ For example, dihydropyridines are reported to blunt spine loss in PD models,^43^, ^103^ and memantine was found to be beneficial in attenuating LID in the animal model.^104^

## Author Roles

1) Research project: A. Conception, B. Organization, C. Execution; 2) Statistical Analysis: A. Design, B. Execution, C. Review and Critique; 3) Manuscript: A. Writing of the first draft, B. Review and Critique.

H.N.: 1A, 1B, 1C, 3A

T.U.: 1B, 1C, 3B

Y.F.: 1B, 1C, 3B

S.U.: 1A, 3B

M.T.: 1A, 1B, 1C, 3B

## Full financial disclosures for the preceding 12 months

The authors have nothing to report.
